# Authentication of Three Source Spices of *Arnebiae Radix* Using DNA Barcoding and HPLC

**DOI:** 10.3389/fphar.2021.677014

**Published:** 2021-07-01

**Authors:** Haiyan Xu, Ping Li, Guangxi Ren, Yanjiao Wang, Dan Jiang, Chunsheng Liu

**Affiliations:** ^1^ School of Chinese Materia Medica, Beijing University of Chinese Medicine, Beijing, China; ^2^ College of Traditional Chinese Medicine, Xinjiang Medical University, Xinjiang, China; ^3^ Department of Basic Medical Sciences, Xinjiang Medical University, Xinjiang, China

**Keywords:** *Arnebiae Radix*, DNA barcoding, ITS2, HPLC, identification

## Abstract

*Arnebia decumbens* (Vent.) Coss. et Kralik*, A. euchroma* (Royle) Johnst and *A. guttata* Bunge, three commonly used traditional Chinese medicinal plants have been widely used for the clinical treatment of inflammatory diseases caused by fungal, bacterial, oxidation, and other related pathogens. However, precise identification at the similar species level is usually challenging due to the influence of the source of medicinal materials, traditional ethnic medicine and medicinal habits. Here we developed a comprehensive and efficient identification system for three source spices of *Arnebiae Radix* based on DNA barcoding and HPLC fingerprinting. A total of 599 samples from thirty-five wild populations were collected and identified by using DNA barcodes of ITS2 regions, and the chemotypes of seven naphthoquinoneswere revealed by HPLC quantitative analysis including principal component analysis and hierarchical clustering analysis. Our results showed that the ITS2 sequences can distinguish three source spices of *Arnebiae Radix* from adulterants. However, it was difficult to identify them by HPLC-specific chromatograms combined with chemometric analysis. These results indicated that DNA barcoding was a more powerful method than HPLC fingerprinting for the identification of related species that were genetically similar. DNA barcoding analysis could be a promising and reliable tool to accurately confirm the identities of medicinal materials, especially for those whose sources are multiple and difficult to be identified by conventional chromatography.

## Introduction

*Arnebiae Radix* (Zicao in Chinese), a kind of traditional Chinese medicine, is the dried root bark of *A*. *euchroma* (Royle) Johnst. and *A*. *guttata* Bunge in the Chinese Pharmacopoeia (2020 version). Shikonin and its derivatives, red naphthoquinones, are widely found in the epidermis of the roots of *Arnebiae Radix* (Zhan et al., 2015) and have been widely demonstrated to possess various biological activities, such as anti-inflammatory (Fu et al., 2016; Sun et al., 2017; Guo et al., 2019), antibacterial (Zhao et al., 2017; Huang et al., 2020), and antiangiogenic (Liu C. et al., 2020) activities. Recently, it was reported that shikonin and its derivatives could induce apoptosis of many types of cancer cells and exhibit anticancer activities and antitumorigenic properties (Liao et al., 2020). *Amebia Radix* has been widely used in the medicine, printing and dyeing industry, cosmetics and food industries (Xu et al., 2014; Ma et al., 2021).

Furthermore, there are other plants of the genus *Arnebia* (Boraginaceae) that are also named Zicao in Xinjiang of China, such as *A. decumbens* (Vent.) Coss. et Kralik and *A. tschimganica* (Fedtsch.) G. L. Chu (Jia-Xin et al., 2018). The market for *Arnebiae Radix* is complicated due to the influence of the source of medicinal materials, traditional ethnic medicine and medicinal habits. Thus, it is difficult to identify the authenticity of medicinal *Arnebiae Radix*, and the identification problem needs to be solved urgently.

Currently, DNA barcoding has emerged as an effective tool for the identification of traditional Chinese medicine due to its species specificity. DNA barcoding has been performed to recognize animals, plants, and fungi (Chen et al., 2014; Gunnels et al., 2020; Behrens-Chapuis et al., 2021; Selva Pandiyan et al., 2020). As a valuable tool for biological identification, DNA barcoding can identify species efficiently and conveniently (Yu et al., 2021). Chen et al. found that the internal transcribed spacer 2 (ITS2) region can potentially be used as a standard DNA barcode to identify medicinal plants and their closely related species (Chen et al., 2010). ITS2 can serve as a novel universal barcoding for the identification of a broader range of plant taxa (Liu et al., 2012; Zhang et al., 2018; Khan et al., 2019). Moreover, high-performance liquid chromatography (HPLC) specific chromatograms, which can effectively determine the content of compounds, are widely used for authenticity confirmation and quality control of traditional Chinese medicines (Hu et al., 2020; Liu B. et al., 2020). Some researchers have indicated that the chemical components of different *Arnebiae Radix* vary, some of which may have good bioactivities (Feng et al., 2020; Liao et al., 2020; Mei et al., 2020).

Thus, authenticity assurance is crucial for their quality control. In this study, we intended to use the DNA barcoding technique and HPLC-specific chromatograms to identify three source spices of *Arnebiae Radix*. The results will facilitate exploring the genetic basis of chemical variations and developing strategies for the utilization and conservation of *Arnebiae Radix*.

## Materials and Methods

### Plant Materials

A total of 599 samples from thirty-five wild populations were collected and analyzed in this study (Table 1), including *A. decumbens* (Vent.) Coss. et Kralik (Ad:120 individuals from eight wild populations), *A. euchroma* (Royle) Johnst (Ae: 227 individuals from 13 wild populations), and *A. guttata* Bunge (Ag: 252 individuals from 13 wild populations). This study contained most of the *Arnebiae radix* species in Taiwan and China but did not include *C. quinquesecta*, because the species is a critically endangered medicinal plant and was not found in the field. Sampling from plantation populations or within short distances was avoided (>50 km). All samples were dried and stored immediately in silica gel after collection. Voucher specimens were deposited at Xinjiang Medical University. The geographic localities of each sampled population were determined using a Garmin GPS unit (Table 1).

**TABLE 1:** Sample information of *Arnebiae Radix* and its adulterants in this study.

Taxon	Sample	Locality	Longitude(E)	Latitude(N)	Altitude(m)	Sample Size
*A. decumbens* (vent.) coss. et kralik	Ad(FK)	Fukang city	88°17′34″	44°24′39″	487	18
	Ad(TKS)	Tekesi county	81°54′41.78″	43°12′10.64″	1375	16
	Ad(MQ)	Miquan county	87°26′47″	44°36′42″	707	15
	Ad(KLMY)	Kelamayi city	84°57′40″	45°11′48″	436	18
	Ad(WS)	Wusu county	84°57′40″	45°11′48″	379	15
	Ad(SHZ)	Shihezi city	86°14′28″	45°1′42″	472	15
	Ad(BEJ)	Buerjin county	86°92′34.51″	47°07′33.08″	497	11
	Ad(SHW)	Shawan county	85°55′17″	44°55′52″	564	12
	Ae(WLMQ)	Wulumuqi county	87°07′28.67″	43°17′19″	2,507	14
	Ae(ML)	Mulei county	90°31′09.79″	43°33′21.42″	2,568	5
	Ae(BCH)	Baicheng county	81°84′55.37″	41°82′11.06″	2,606	20
	Ae(NLT)	Nalati town	83°56′10.920″	43°10′11.24″	2,500	16
	Ae(TSHKEG)	Tashikuergan county	75º04′52.2″	37º49′58.7″	4,234	18
*A. euchroma* (royle) Johnst	Ae(ATSH)	Atushi county	76°12′33.08″	39°27′43.50″	2,300	20
	Ae(WQ)	Wenquan county	80°32′18″	45°2′18″	2,299	19
	Ae(HJ)	Hejing county	84º07′13.3″	42º42′00.5″	2,456	19
	Ae(HCH)	Huocheng county	81°09′53.83″	44°27′32.39″	2,502	21
	Ae(JH)	Jinghe county	83º15′44.7″	44º23′42.6″	2,144	19
	Ae(GL)	Gongliu county	82°23′19.10″	43°35′42.30″	2,530	19
	Ae(AKS)	Akesu county	80°29′31.22″	41°15′33.28″	2030	19
	Ae(XY)	Xinyuan county	83°27′66.19″	43°41′55.40″	2,201	18
	Ag(HM)	Hami county	93°50′53.05″	43°07′42.63″	861	18
	Ag(FY)	Fuyun county	89°1′2″	45°2′46″	1065	22
	Ag(BLK)	Balikun county	91°39′48.60″	43°47′25,6″	1632	17
	Ag(NLK)	Nileke county	82°10′3″	43°36′41″	765.4	16
	Ag(TSHKEG)	Tashikuergan county	75º28′58.3″	37º13′44.7″	3,780	14
	Ag(QT)	Qitai county	091°22′52.9″	44°58′0.5″	1167	19
	Ag(HJ)	Hejing county	86°0′23.56″	43°01′1.89″	2,253	17
*A. guttata* bunge	Ag(YW)	Yiwu county	94°48′51.30″	43°19′25.3″	1414	19
	Ag(SHSH)	Shanshan county	89°56′37.05″	43°06′2,0″	653	15
	Ag(TL)	Tuoli county	82°34′40″	45°35′0″	1532.3	17
	Ag(ML)	Mulei county	091°23′22.5″	45°03′20″	1312	23
	Ag(QH)	Qinghe county	90°22′25″	45°33′7″	1193	20
	Ag(WQ)	Wenquan county	81°8′18″	44°46′32″	1801.7	16
	Ag(XY)	Xinyuan county	82°29′45″	43°24′17″	894.1	19

### Chemical Apparatus

Chemical standards including (β,β-dimethylacryl)shikonin (15102821), alkannin (15102721), deoxyalkannin (15062422) and acetylshikonin (15120431) were purchased from Tauto Biotech (Shanghai, China). β-Acetoxyisovalerylalkannin (P05M7F14235) was purchased from Yuanye Biotech (Shanghai, China). Isobutylshikonin (wkq16101302) was purchased from Weikeqi Biotech (Sichuan, China). (2-Methyl-n-nbutyl) shikonin (AV51-LDQR) was purchased from Tokyo Chemical Industry (Tokyo, Japan). The purity of the standards was above 98%. The petroleum ether (60–90°C) was analytically pure. All of the chemicals and reagents used in this study were of HPLC analytical grade.

### DNA Extraction, PCR Amplification and DNA Sequencing

The material specimens were dried by natural methods, and 20 mg of dried plant material was used for DNA extraction. Genomic DNA was extracted with a DNA Secure Plant Kit from Tiangen Biotech (Beijing, China). The relative purity and concentration of extracted DNA were estimated by ethidium bromide staining on agarose gels and compared with known DNA concentration markers.

The extracted genomic DNA was amplified by polymerase chain reaction (PCR), using the ITS2 (ITS2F, 5′-ATG​CGA​TAC​TTG​GTG​TGA​AT-3′ and ITS2R, 5′-GAC​GCT​TCT​CCA​GAC​TAC​AAT-3′). PCR amplifications were carried out in a volume of 20 μL using 1 μL of template DNA (50–100 ng), 2 μL of 10 × reaction buffer, 1.6 μL of dNTP mix (2.5 mM), 1.25 μL of 10 μM of each primer, 0.2 μL of Ex-Taq DNA polymerase (Takara Shuzo Co., Ltd., Otsu, Japan), and 12.7 μL of sterile distilled water. Reactions were run on a Veriti thermocycler (Applied Biosystems, United States). The PCR conditions for amplification consisted of one cycle of denaturation at 95°C for 5 min, 35 cycles of 1 min of denaturation at 94°C, 1 min of annealing at 55°C and 1 min 30 s of extension at 72°C, followed by an 8 min extension step at 72°C. PCR products were electrophoresed on 1.5% (w/v) agarose gels and purified through precipitation with 95% ethanol and 3 M sodium acetate (pH 5.2). All the purified PCR products were sequenced directly in both directions on an ABI 3730XL automated sequencer (Applied Biosystem, Foster City, CA, United States).

### DNA Barcoding Analysis

Cutting and splicing of all ITS2 sequences, removal of the primer region and low-quality regions, manual correction, and stitching were performed by ContigExpress software. Then, the modified ITS2 sequences were submitted to DNAMAN software to compare the similarities of the samples. Finally, the modified ITS2 sequences were aligned, and the inter/intraspecific genetic distances were measured using MEGAX 10.2.4 software. A phylogenetic tree using GenBank sequences as outgroups was constructed based on standard parameters with bootstrap testing of 1,000 replicates.

### HPLC Conditions

The medicinal powder precisely was weighed to approximately 1 g and placed in an Erlenmeyer flask; 50 ml of petroleum ether (60–90°C) was added. Then, the sample was accurately weighed and extracted with 30 min ultrasonication steps. After cooling and adding petroleum ether (60–90°C) to compensate for the decrease in weight, the sample was filtered. The resulting filtrate was measured (10 ml of *A. euchroma*, 30 ml of *A. guttata* Bunge, 30 ml of *A. tschimganica*, and 30 ml of *A. decumbens* (Vent.) Coss. et Kralik), evaporated to dryness, dissolved the residue in acetonitrile, transferred to a 10 ml volumetric flask, and dissolved in acetonitrile. The sample was then transferred to a 10 ml volumetric flask, diluted with acetonitrile to scale, shaken to mix well and prepared for analysis. HPLC chromatographic conditions were conducted as described by Ding et al. (Ding et al., 2019).

### Chemometric Analysis

The reference chromatogram was generated using a Similarity Evaluation System for Chromatographic Fingerprint of TCM (Version 2012). Principal component analysis (PCA) and hierarchical clustering analysis (HCA) were performed by the professional software SIMCA 14.1 to demonstrate the variability of the relative peak areas of bioactive compounds. Outliers were identified and removed by DmodX.

## Results

### Genetic Divergence Determination

**FIGURE 1:**
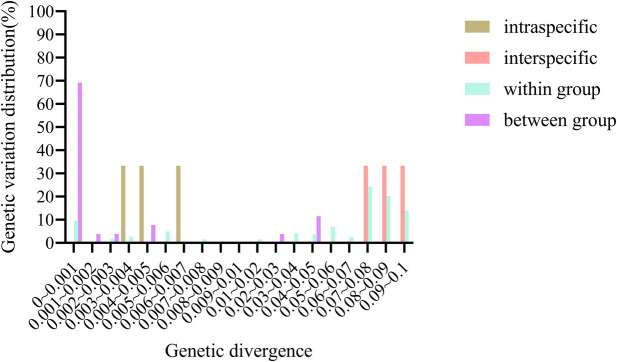
Genetic divergence of the ITS2 region of the three *Arnebiae Radix* species.

The length of the aligned ITS2 sequences was 399 bp, and the number of variable sites was 71 (Table 2). We used four parameters to characterize divergence. The intraspecific distance of ITS2 was 0.0025–0.006, and the interspecific distance was 0.0745–0.0915. Additionally, after grouping samples according to locality, the genetic distance within a group was 0–0.0494, and the genetic distance between groups was 0–0.0941. The analysis of the distribution of genetic distance (Figure 1) showed obvious barcoding gaps between samples, indicating that the ITS2 sequence has a strong ability to identify *Arnebia* genus samples at the species level.

**TABLE 2:** Variable sites of the ITS2 region of the three *Arnebiae Radix* species.

Site	Ad	Ae	Ag
1	−/A	−/A	−/A/G
2	−/A	−/G	−/A/G
13	C	C	C/T
26	C/T	C	C
40	C	C/T	C/T
41	G	A	A/G
42	C	C/T	C
44	T	T	A/T
45	C	C/T	A/C
46	G	C/G	G/T
52	C	C	C/T
60	C	A	A/C
63	A	A	A/T
64	G	A	A/G
66	A	A	A/G
67	C	A/C	A
68	T	T	C/T
69	T	G	G/T
70	G	T	A/T
71	C	T	T
77	T	G/T	C/T
98	G	G	A/G
101	C	T	T
102	C/T	T	T
103	G	G	G/T
104	G	A	A/T
118	A/G	G	A/G
122	A/T	T	T
124	C/T	C	C/T
130	C/T	C	C/T
137	G/T	T	G/T
149	A/G/T	A	A/T
180	A/C	A	A/C
184	C/T	C/T	C
194	A/G	A/G	A
200	A/G	G	A/G
201	C/T	T	T
202	G/T	G	G/T
203	C/G/T	T	C/T
207	C/T	C	C/T
208	C/G	G	G
210	A/G	A	A/G
218	A/T	A	A
219	G/T	G/T	G/T
223	C/T	C	C/T
226	G/T	G	G/T
227	C/T	C	C/T
231	C/T	C	C/T
232	A/C	C	A/C
233	A/G	A	A
236	C/T	T	C/T
238	G/T	G	G/T
239	C/T	T	T
240	G/T	T	G/T
241	C/G/T	C	C/T
252	C/T	C	C/T
254	G/T	G	G/T
261	C/G	C/G	G
263	C/T	C	C/T
264	C/T	C	C/T
267	C/G	C/G	G
286	C/T	T	C/T
348	A/G	A/G	G
359	C/T	C	C
371	A/T	A	A/T
380	−/G/T	G/T	−/T
389	−/A/G	G	−/G
390	−/A/G	G	−/A/G
391	−/C/T	C	−/C/T
396	−/C/T	C/T	−/C/T
399	−/G/T	−/T	−/G/T

Ad, *A. decumbens* (Vent.) Coss. et Kralik; Ae, *A. euchroma* (Royle) Johnst; Ag, *A. guttata* Bunge; -missing variant site.

### Identification of *Arnebiae Radix* by DNA Barcoding

**FIGURE 2:**
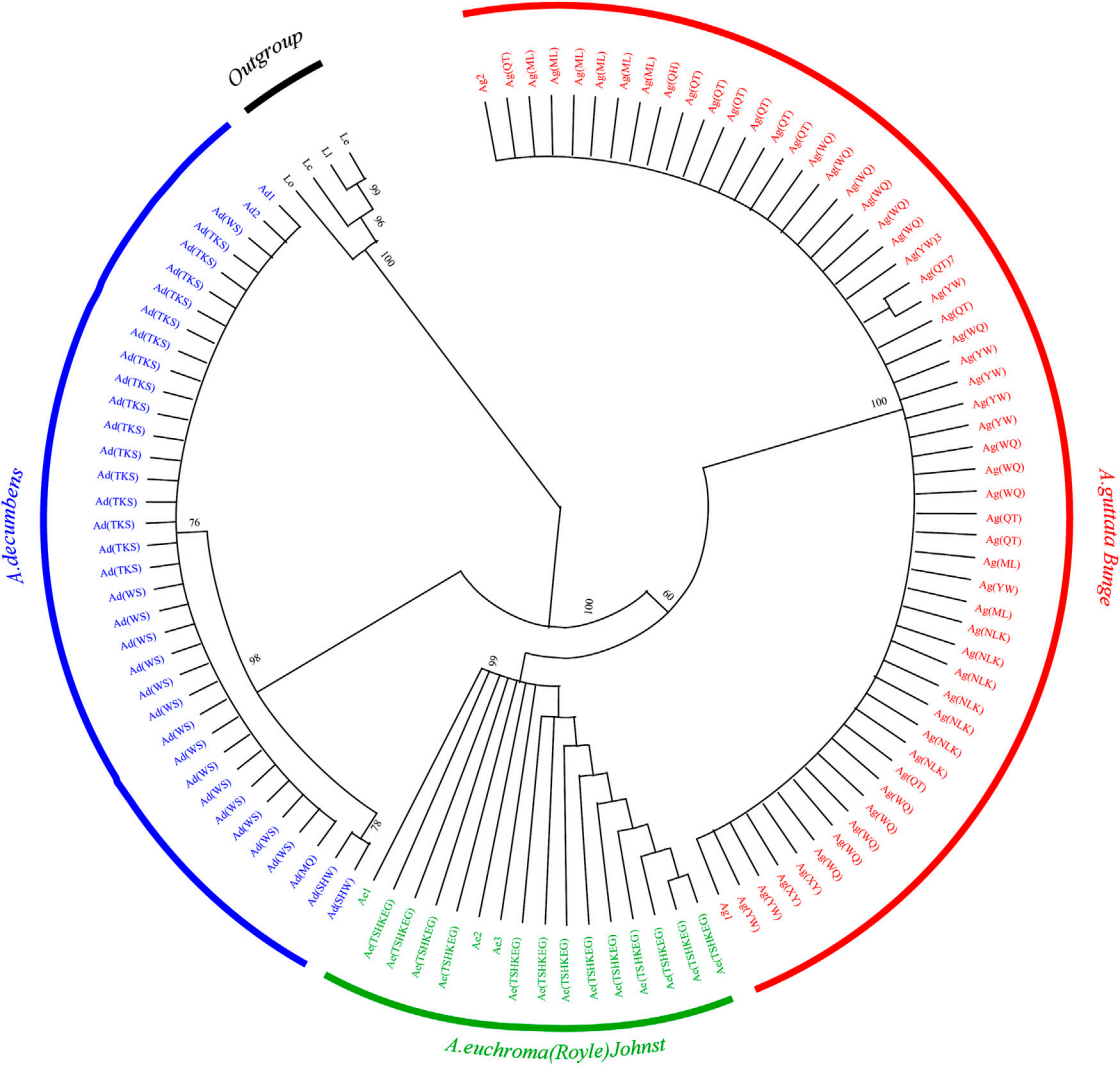
Results of the neighbor-joining tree based on ITS2 sequences of the 599 *Arnebiae Radix* samples. Supporting values (>50%) are indicated above or below the relevant branches. Some subtrees are compressed to show the samples completely in a picture. Ad, *A. decumbens* (Vent.) Coss. et Kralik; Ae, *A. euchroma* (Royle) Johnst; Ag, *A. guttata* Bunge. Ad1:17 samples of Ad(FK),18 samples of Ad(KLMY),nine samples of Ad(SHW) and 15 samples of Ad(SHZ); Ad2:11 samples of Ad(BEJ),14 samples of Ad(MQ),one samples of Ad(SHW), and one sample of Ad(FK);Ae1:19 samples of Ae(WQ), 14 samples of Ae(WLMQ), 19 samples of Ae(HJ), 16 samples of Ae(NLT),15 samples of Ae(XY), 20 samples of Ae(JH), 21 samples of Ae(HCH), 19 samples of Ae(GK) and 20 samples of Ae(ATSH);Ae2: six samples of Ae(TSHKEG);Ae3: five samples of Ae(ML) ,20 samples of Ae(BCH) and 19 samples of Ae(AKS);Ag1: nine samples of Ag(NLK), and 17 samples of Ag(XY);Ag2: four samples of Ag(TL), 14 samples of Ag(HJ), three samples of Ag(SHSH), and one samples of Ag(TSHKEG).

To identify the species of the 599 *Arnebiae Radix* samples more accurately and visually, we constructed a neighbor-joining tree based on the ITS2 sequences obtained from the samples and four ITS2 sequences of Boraginaceae downloaded from NCBI (Supplementary Table S1). The ITS2 sequences were divided into mutually exclusive monophyletic clades. *A. guttata* Bunge clustered into one subgroup, *A. euchroma* (Royle) Johnst could be clustered into one group, and *A. decumbens* (Vent.) Coss. et Kralik could be clustered into one group (Figure 2). The pairwise distance analysis supports this interpretation, revealing that ITS2, as a barcode, is able to distinguish between the three species of *Arnebiae Radix*.

### HPLC Fingerprint of *Arnebiae Radix* Samples

The results of the determination of seven naphthoquinones in the roots of three species of *Arnebiae Radix* from different habitats in Xinjiang are shown in Table 3. Naphthoquinones were found in the roots of all three *Arnebiae Radix* species, and there were differences among different species. The total amount of naphthoquinones in Ae was the highest (43.3505 mg/g), followed by that in Ag (11.4042 mg/g), and the lowest amount (6.0462 mg/g) was found for Ad. It was assumed that Ad was an annual herb, the rest were perennial herbs, and the accumulation of each component in plants was different. Ag and Ae both contain seven components, while β-acetoxyisovaleryl acarnin β and β'-dimethylacrylamine were not detected in Ad. According to the Chinese Pharmacopoeia (2020 edition), the content of β,β'-dimethylacrylamine in *Arnebiae Radix* should not be less than 0.30%. This study found that this component in Ae and Ag met the pharmacopoeia requirements. This study will provide a reference basis for exploring new drug sources.

**TABLE 3:** Content of seven bioactive components of the 35 *Arnebiae Radix* samples. (mg/g).

	L-shikonin	Acetylshikonin	β-Acetoxyisovaleryl acarnin	Deoxyshikonin	Isobutyryl shikonin	β. β′-dimethylacrylamine	2-methylbutyl Shikonin	Total
Ag(HM)	−	0.69	1.0	−	−	0.84	1.5	4.0
Ag(FY)	−	0.46	0.74	−	−	0.67	1.1	3.0
Ag(BLK)	−	1.1	0.87	0.26	−	0.65	1.2	4.1
Ag(NLK)	0.10	1.6	3.0	0.19	0.38	0.73	2.4	8.4
Ag(TSHKEG)	0.57	8.1	9.8	0.58	2.0	1.3	11	34
Ag(QT)	0.12	2.2	1.3	0.29	−	1.6	1.9	7.3
Ag(HJ)	0.11	1.6	3.2	0.21	0.55	0.95	4.0	11
Ag(YW)	−	0.96	1.0	0.23	−	0.82	1.4	4.5
Ag(SHSH)	0.94	2.9	14	0.21	1.8	1.33	15	36
Ag(TL)	−	1.3	1.2	−	−	0.44	1.0	4.0
Ag(ML)	0.15	3.3	0.81	0.23	−	0.55	1.1	6.1
Ag(QH)	0.14	1.5	1.6	0.26	−	1.31	2.1	6.9
Ag(WQ)	−	0.61	1.2	−	−	0.42	0.72	3.0
Ag(XY)	0.13	4.0	3.5	0.49	0.62	1.33	5.7	16
Average	0.28	2.2	3.1	0.29	1.1	0.92	3.6	11
Ae(WLMQ)	0.23	4.4	1.9	−	1.7	4.6	7.9	21
Ae(ML)	0.21	2.8	1.9	−	1.4	3.0	7.1	16
Ae(BCH)	0.25	2.7	1.3	0.23	1.2	3.0	6.6	15
Ae(NLT)	0.68	22	3.6	0.67	6.0	11	32	76
Ae(TSHKEG)	0.19	1.4	8.4	−	3.2	0.60	4.1	18
Ae(ATSH)	0.67	9.1	2.7	0.30	2.9	5.5	14	35
Ae(WQ)	1.07	9.4	3.7	0.37	8.6	8.0	17	48
Ae(HJ)	0.54	18.9	3.1	0.63	4.4	12	24	63
Ae(HCH)	0.68	12.6	5.4	0.36	7.1	4.1	17	47
Ae(JH)	0.75	12.2	4.7	0.69	6.8	5.1	15	46
Ae(GL)	0.80	20.0	4.2	0.64	4.2	14	26	70
Ae(AKS)	0.36	9.0	1.7	−	2.3	4.2	11	29
Ae(XY)	0.84	25.2	3.8	0.90	5.6	13	29	78
Average	0.56	11.5	3.6	0.53	4.2	6.8	16	43
Ad(FK)	−	4.4	−	0.25	0.43	−	3.3	8.4
Ad(TKS)	−	4.1	−	0.93	0.22	−	1.2	6.4
Ad(MQ)	−	1.7	−	0.29	0.14	−	0.81	3.0
Ad(KLMY)	0.13	8.2	−	0.66	0.32	−	2.4	12
Ad(WS)	−	2.5	−	0.21	0.25	−	1.8	4.7
Ad(SHZ)	−	3.1	−	0.42	0.25	−	1.3	5.0
Ad(BEJ)	−	2.4	−	0.20	0.32	−	2.4	5.3
Ad(SHW)	−	1.5	−	0.20	0.22	−	0.92	2.8
Average	0.13	3.5	−	0.39	0.27	−	1.8	6.0

Ad, *A. decumbens*; Ae, *A. euchroma* (Royle) Johnst; Ag, *A. guttata* Bunge.

**FIGURE 3:**
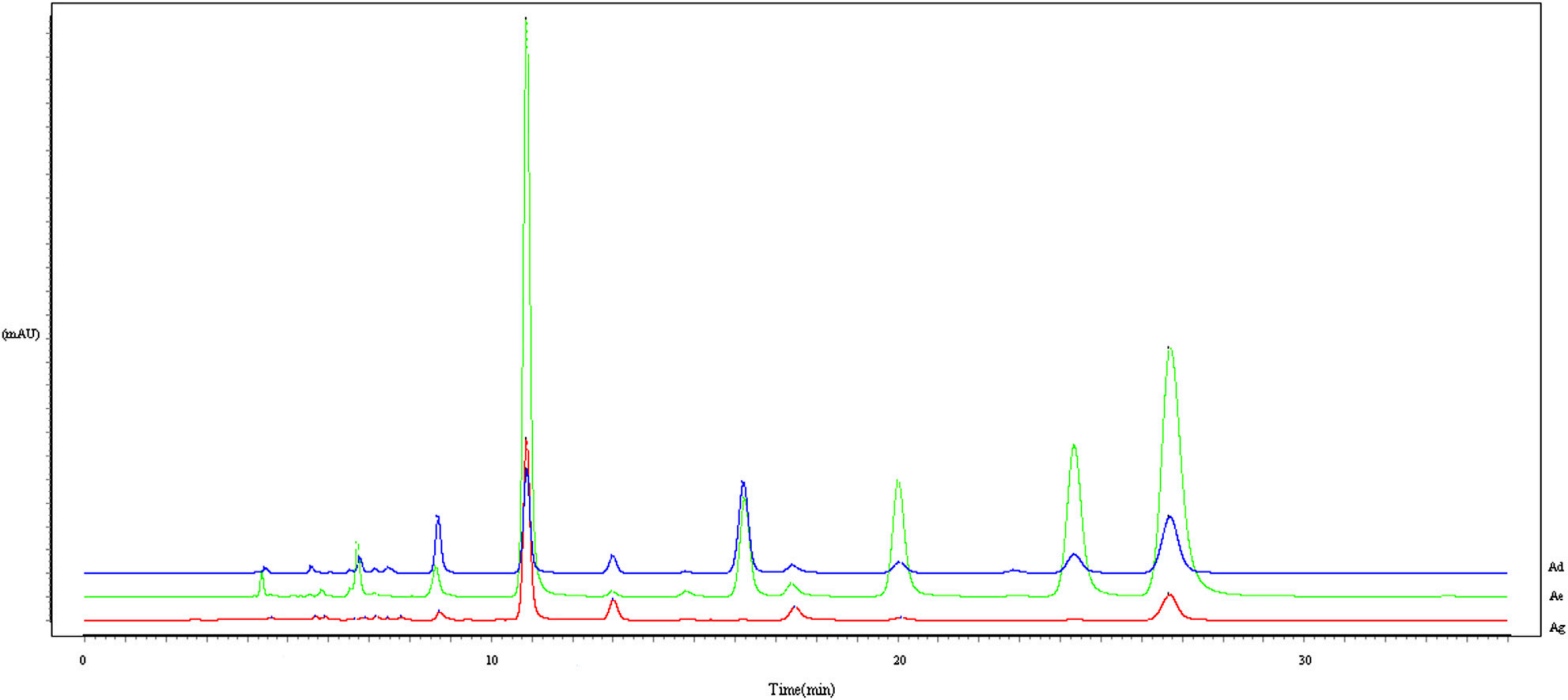
Representative HPLC fingerprints of three *Arnebiae radix* species. Ad, *A. decumbens*; Ae, *A. euchroma* (Royle) Johnst; and Ag, *A. guttata* Bunge.

To establish the chromatographic fingerprint, 35 *Arnebiae Radix* samples from different species were analyzed under the optimized chromatographic analysis conditions. All chromatograms were matched through multipoint correction and free matching, and the bottom sample was the reference sample (Supplementay Figures S1, S2). The representative HPLC fingerprints were so similar that it was difficult to separate the three spaces visually (Figure 3).

### Principal Component Analysis of HPLC Fingerprint of *Arnebiae Radix* Samples

**FIGURE 4:**
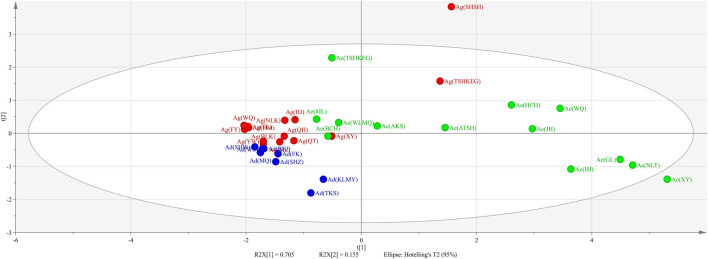
Score plot of PCA of the 35 Arnebiae Radix samples. Ad, *A. decumbens*; Ae, *A. euchroma* (Royle) Johnst; Ag, *A. guttata* Bunge.

PCA, a multivariate method, is widely used in data analysis to summarize variation, and is implemented as a data-reduction technique to generate a visual scatter plot for the qualitative evaluation of similarities and differences within multivariate data. To differentiate all the *Arnebiae Radix* samples clearly, we carried out PCA according using the data for the seven common characteristic peaks. The score plot was structured based on the first three principal components, which accounted for more than 94.3% of the total variability. We discarded the other principal components because they had little effect on the model. The results showed that all samples were divided into six groups according to their different sources (Figure 4). Group 1 contained two samples belonging to Ag, Group 2 contained four samples belonging to Ae, and Group 3 contained four samples belonging to Ae. Group 4 included five samples belonging to Ae, Group 5 included two samples belonging to Ad, and Group 6 included 12 samples belonging to Ag and six samples belonging to Ad. The results were consistent with the HPLC fingerprint analysis. The results of the HPLC-specific chromatograms combined with PCA were not as accurate as those of DNA barcoding.

### Hierachical Cluster Analysis of HPLC Fingerprint of *Arnebiae Radix* Samples

**FIGURE 5:**
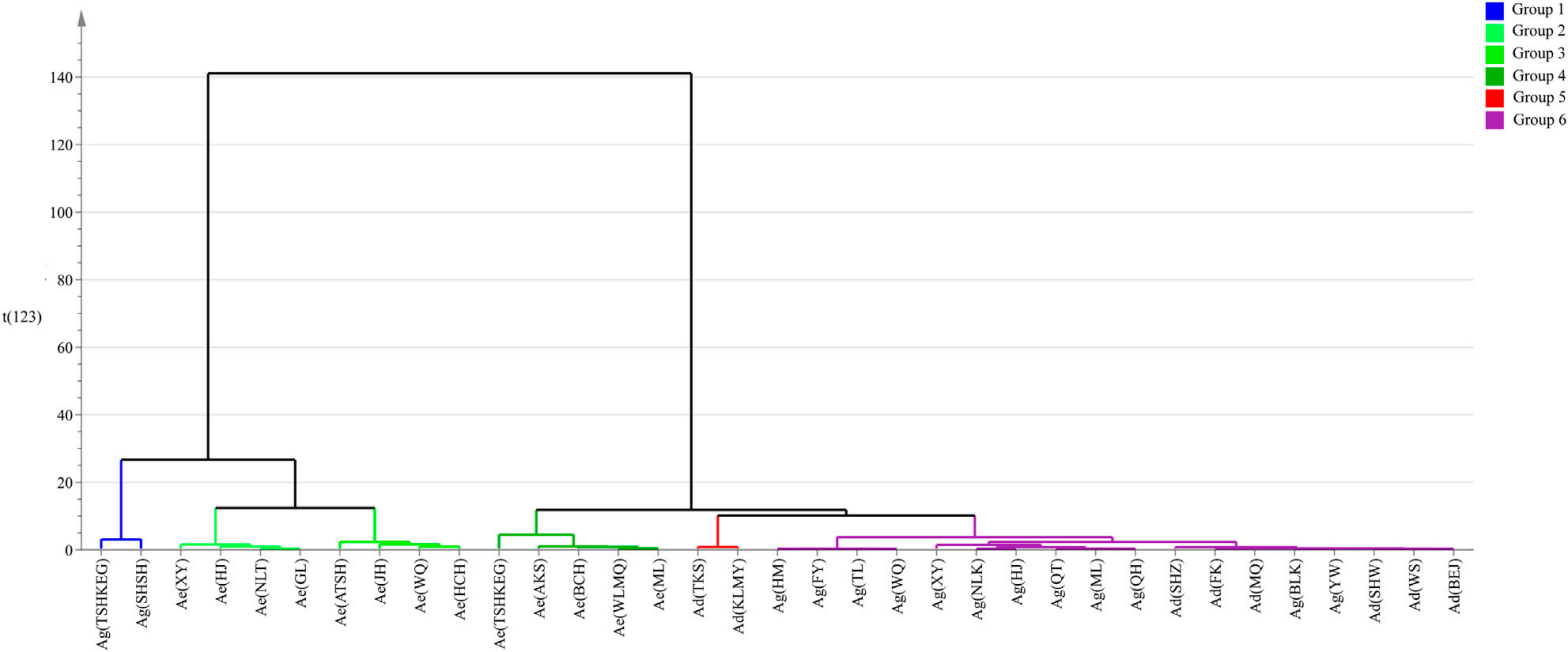
The results of HCA of the 35 *Arnebiae Radix* samples. Calculated with Ward and stored by size. Ad, *A. decumbens*; Ae, *A. euchroma* (Royle) Johnst; and Ag, *A. guttata* Bunge.

HCA is one of the most commonly used unsupervised pattern recognition methods, and is a useful multivariate statistical technique. It can create a cluster tree to assign a data set into groups according to similarity. To show the degree of similarity and difference among the 35 *Arnebiae Radix* samples more clearly, HCA was performed based on the results of PCA of all common characteristic peaks. As shown in Figure 5, all samples were divided into two main clusters according to their similarities and differences. Cluster one included three groups: Group 1 contained two samples belonging to Ag, Group 2 contained four samples belonging to Ae, and Group 3 contained four samples belonging to Ae. Cluster two was divided into three groups: Group 4 included five samples belonging to Ae, Group 5 included two samples belonging to Ad, and Group 6 included 12 samples belonging to Ag and six samples belonging to Ad. Group 4 merged with Group 5 to form a larger branch. All samples in the branch were gathered from Ad, Ae, and Ag. The results of the HPLC-specific chromatograms combined with HCA were not as accurate as those of DNA barcoding.

## Discussion

*Arnebiae Radix*, a commonly used herbal medicine in China, is also widely used in the food and cosmetics industries. As the ecological environment has been constantly destroyed, the wild resources of *Arnebiae Radix* have been sharply reduced, and cultivation is very difficult. There is a serious shortage of *Arnebiae Radix* supplies, leading to a complex and confusing market. The traditional classification method is based on the roots, leaves, flowers, fruits, and other organs of plants. Due to the lack of accurate identification characteristics, the processed commodities only retain the root, which creates great difficulties in the identification of *Arnebiae Radix*. However, the accuracy of the original medicinal materials is required to ensure the effectiveness and safety of clinical medication. DNA barcoding technology techniques are not influenced by organs, growth conditions, tissue differences or the external environment, among other factors (Bhargava and Sharma 2013; Mohammed Abubakar et al., 2017). In this study, the DNA barcoding technique showed the ability to scientifically and accurately identify the species. In the preliminary experimental stage, another three sequences, *matK*, *rbcL*, and ITS, were also considered, but it was found that there were many nested peaks within ITS, and the variable sites measured by *matK* and *rbcL* were not obvious. The established PCR-RFLP method based on the ITS2 sequence can identify *A. euchroma (*Royle) Johnst and *A. guttata* Bunge, as well as other plants also named Zicao in China (Qian et al., 2019). Thus, we examined the ITS2 sequence similarity, genetic distance and phylogenetic tree by using DNA barcoding technology based on its ability to differentiate *Arnebiae Radix* species. Our results suggested that ITS2 can discriminate *A. euchroma* (Royle) Johnst, *A. guttata* Bunge and *A. decumbens* (Vent.) Coss. et Kralik (Figure 2). As one of the most important markers in molecular phylogenetic research, the ITS2 sequence has obvious sequence variation at the species level or subspecies level, and it is an important candidate barcode for identification at the species level or subspecies level (Sickel et al., 2015; Cheng et al., 2016).

HPLC-specific chromatograms were established, and the contents of six hydroxyl naphthoquinones can be used to distinguish the different origins of *Arnebiae Radix* herbs (Ke et al., 2016). Therefore, we utilized HPLC methods to differentiate the three *Arnebiae Radix* species. Our results indicated that this method allows for the simultaneous discrimination of the seven main naphthoquinones in these samples. The lichen *A. euchroma* (Royle) Johnst shows high intraspecific chemical variations in Xinjiang, while *A. guttata* Bunge and *A. decumbens (Vent.) Coss. et Kralik*show relatively less variation (Table 3). This result could partly be explained by the limited distribution of Ag and Ad, resulting in less variation. Ag and Ad have restricted geographic distributions in western and northern Xinjiang, whereas Ae has a rather wide distribution around Xinjiang. However, the samples could not be distinguished based on HPLC fingerprint (Figure 3), PCA (Figure 4) and HCA methods (Figure 5). Ecological factors, especially altitude, may be responsible for this result. Additionally, other environmental factors, such as light, temperature, air, soil, and moisture, also affect the content of the chemical components of plants. To further develop and utilize the plant resources of *Arnebiae Radix*, it is necessary to study more about the how genetic and environmental factors influence the metabolites of *Arnebiae Radix*.

In summary, this study has established a system for identifying the three *Arnebiae Radix* species based on DNA barcoding and chemical analysis methods. The results revealed that although the HPLC method cannot differentiate these samples, DNA barcoding can transcend the limitations of HPLC methods to ensure effective and universal proof of medicinal plants from different species. Similar results showed that DNA barcoding was a promising and reliable tool for the identification of three kinds of *Plumeria* flowers compared to HPLC-specific chromatograms, which are generally used (Zhao et al., 2018). Thus, DNA barcoding is more powerful than HPLC fingerprinting for species traceability in related species that are genetically similar. Our findings may be useful for the determination of naphthoquinones of *Arnebiae Radix* and provide a reference for the identification of traditional Chinese medicine based on DNA barcoding. Due to the narrow distribution of *A. tschimganica* (Fedtsch.) G. L. Chu, no sample was used in this experiment. It is necessary to expand the sample size and investigate the corresponding response of different growth periods and growing environments to provide a reference for the quality control and expansion of new drug sources of *Arnebiae Radix.*

## Data Availability

The datasets presented in this study can be found in online repositories. The names of the repository/repositories and accession number(s) can be found in the article/Supplementary Material.

## References

[B1] Behrens-Chapuis S. Herder F. Geiger M. F. (2021). Adding DNA Barcoding to Stream Monitoring Protocols - What's the Additional Value and Congruence between Morphological and Molecular Identification Approaches? PLoS One 16 (1), e0244598. 10.1371/journal.pone.0244598 33395693 PMC7781668

[B2] Bhargava M. Sharma A. (2013). DNA Barcoding in Plants: Evolution and Applications of In Silico Approaches and Resources. Mol. Phylogenet. Evol. 67 (3), 631–641. 10.1016/j.ympev.2013.03.002 23500333

[B3] Chen S. Pang X. Song J. Shi L. Yao H. Han J. (2014). A Renaissance in Herbal Medicine Identification: from Morphology to DNA. Biotechnol. Adv. 32 (7), 1237–1244. 10.1016/j.biotechadv.2014.07.004 25087935

[B4] Chen S. Yao H. Han J. Liu C. Song J. Shi L. (2010). Validation of the ITS2 Region as a Novel DNA Barcode for Identifying Medicinal Plant Species. PLoS One 5 (1), e8613. 10.1371/journal.pone.0008613 20062805 PMC2799520

[B5] Cheng T. Xu C. Lei L. Li C. Zhang Y. Zhou S. (2016). Barcoding the Kingdom Plantae: New PCR Primers forITSregions of Plants with Improved Universality and Specificity. Mol. Ecol. Resour. 16 (1), 138–149. 10.1111/1755-0998.12438 26084789

[B6] Ding W. H, Yan X. H Ge L. Xu H. Y. (2019). Determination of Naphthoquinones in Roots of Four Species of Arnebia in Xinjiang by HPLC. Chin. Traditional Patent Med. 41 (04), 936–939. 10.3969/j.Issn.1001-1528.2019.04.047

[B7] Feng J. Yu P. Zhou Q. Tian Z. Sun M. Li X. (2020). An Integrated Data Filtering and Identification Strategy for Rapid Profiling of Chemical Constituents, with Arnebiae Radix as an Example. J. Chromatogr. A 1629, 461496. 10.1016/j.chroma.2020.461496 32846341

[B8] Fu D. Shang X. Ni Z. Shi G. (2016). Shikonin Inhibits Inflammation and Chondrocyte Apoptosis by Regulation of the PI3K/Akt Signaling Pathway in a Rat Model of Osteoarthritis. Exp. Ther. Med. 12 (4), 2735–2740. 10.3892/etm.2016.3642 27703516 PMC5038895

[B9] Gunnels T. Creswell M. McFerrin J. Whittall J. B. (2020). The ITS Region Provides a Reliable DNA Barcode for Identifying Reishi/lingzhi (Ganoderma) from Herbal Supplements. PLoS One 15 (11), e0236774. 10.1371/journal.pone.0236774 33180770 PMC7660467

[B10] Guo H. Sun J. Li D. Hu Y. Yu X. Hua H. (2019). Shikonin Attenuates Acetaminophen-Induced Acute Liver Injury via Inhibition of Oxidative Stress and Inflammation. Biomed. Pharmacother. 112, 108704. 10.1016/j.biopha.2019.108704 30818140

[B11] Hu L. Zhou C. Huang Y. C. Wang Y. Wei G. Liang Z. (2020). HPLC Coupled with Electrospray Ionization Multistage MS/MS and TLC Analysis of flavones‐C‐glycosides and Bibenzyl of Dendrobium Hercoglossum. J. Sep. Sci. 43 (20), 3885–3901. 10.1002/jssc.202000647 32803831

[B12] Huang X. Y. Fu H. L. Tang H. Q. Yin Z. Q. Zhang W. Shu G. (2020). Optimization Extraction of Shikonin Using Ultrasound-Assisted Response Surface Methodology and Antibacterial Studies. Evid. Based Complement. Alternat Med. 2020, 1208617. 10.1155/2020/1208617 32802111 PMC7411493

[B13] Jia-Xin T. F. G. Zhi-Lai Z. (2018). Herbalogical Textual of Arnebiae Radix and Analysis of Medicinal Resources. Mod. Chin. Med. 20 (9), 1064–1067. 10.13313/j.issn.1673-4890.20180428002

[B14] Ke Z. Rui S. Aijun T. Hong W. Jie L. Linong G. (2016). HPLC Specific Chromatogram of Arnebia Euchroma and Determination of Six Naphthoquinones in Boraginaceous Herbs. Chin. J. Pharm. Anal. 36 (9), 1526–1535. 10.16155/j.0254-1793.2016.09.03

[B15] Khan S. A. Baeshen M. N. Ramadan H. A. Baeshen N. A. (2019). ITS2: An Ideal DNA Barcode for the Arid Medicinal Plant Rhazya Stricta. Pharm. Med. 33 (1), 53–61. 10.1007/s40290-019-00266-3 31933272

[B16] Liao M. Yan P. Liu X. Du Z. Jia S. Aybek R. (2020). Spectrum-effect Relationship for Anti-tumor Activity of Shikonins and Shikonofurans in Medicinal Zicao by UHPLC-MS/MS and Chemometric Approaches. J. Chromatogr. B 1136, 121924. 10.1016/j.jchromb.2019.121924 31841980

[B17] Liu B. Hu T. Yan W. (2020). Authentication of the Bilberry Extracts by an HPLC Fingerprint Method Combining Reference Standard Extracts. Molecules 25 (11), 2514. 10.3390/molecules25112514 PMC7321295 32481617

[B18] Liu C. He L. Wang J. Wang Q. Sun C. Li Y. (2020). Anti-angiogenic Effect of Shikonin in Rheumatoid Arthritis by Downregulating PI3K/AKT and MAPKs Signaling Pathways. J. Ethnopharmacology 260, 113039. 10.1016/j.jep.2020.113039 32497675

[B19] Liu Z. Zeng X. Yang D. Ren G. Chu G. Yuan Z. (2012). Identification of Medicinal Vines by ITS2 Using Complementary Discrimination Methods. J. Ethnopharmacology 141 (1), 242–249. 10.1016/j.jep.2012.01.057 22353709

[B20] Ma S. J. Geng Y. Ma L. (2021). Advances in Studies on Medicinal Radix Arnebia. Mod. Chin. Med. 23 (01), 177–184. 10.13313/j.issn.1673-4890.20190719004

[B21] Mei L. Ling-Feng W. Hong-Liang J. (2020). Reviews on Natural Quinones and Their Bioactivities of Medicinal Zicao. Nat. Product. Res. Dev. 32 (04), 694–707. 10.16333/j.1001-6880.2020.4.021

[B22] Mohammed Abubakar B. Mohd Salleh F. Shamsir Omar M. S. Wagiran A. (2017). Review: DNA Barcoding and Chromatography Fingerprints for the Authentication of Botanicals in Herbal Medicinal Products. Evid. Based Complement. Alternat Med. 2017, 1352948. 10.1155/2017/1352948 28536641 PMC5425840

[B23] Qian L. LIu J. Guo L. Zheng J. I. Ma S. (2019). Molecular Identification of Arnebiae Radix by PCR-RFLP Based on the ITS2 Sequence. Chin. J. Pharm. Anal. 36 (9), 1611–1617. 10.16155/j.0254-1793.2016.09.14

[B24] Selva Pandiyan A. Siva Ganesa Karthikeyan R. Rameshkumar G. Sen S. Lalitha P. (2020). Identification of Bacterial and Fungal Pathogens by rDNA Gene Barcoding in Vitreous Fluids of Endophthalmitis Patients. Semin. Ophthalmol. 35, 358–364. 10.1080/08820538.2020.1864416 33390091

[B25] Sickel W. Ankenbrand M. J. Grimmer G. Holzschuh A. Härtel S. Lanzen J. (2015). Increased Efficiency in Identifying Mixed Pollen Samples by Meta-Barcoding with a Dual-Indexing Approach. BMC Ecol. 15, 26194794. 10.1186/s12898-015-0051-y PMC4509727 26194794

[B26] Sun W. X. Liu Y. Zhou W. Li H. W. Yang J. Chen Z. B. (2017). Shikonin Inhibits TNF-Alpha Production through Suppressing PKC-NF-kappaB-dependent Decrease of IL-10 in Rheumatoid Arthritis-like Cell Model. J. Nat. Med. 71 (2), 349–356. 10.1007/s11418-016-1064-3 27943122

[B27] Xu C. M. Li D. Wang Y. Y. (2014). Research Development of Food Pigment Biosynthesis. J. Chin. Inst. Food Sci. Technol. 14 (02), 225–233. 10.16429/j.1009-7848.2014.02.046

[B28] Yu J. Wu X. Liu C. Newmaster S. Ragupathy S. Kress W. J. (2021). Progress in the Use of DNA Barcodes in the Identification and Classification of Medicinal Plants. Ecotoxicol. Environ. Saf. 208, 111691. 10.1016/j.ecoenv.2020.111691 33396023

[B29] Zhan Z. L. Hu J. Liu T. Kang L. P. Nan T. G. Guo L. P. (2015). Advances in Studies on Chemical Compositions and Pharmacological Activities of Arnebiae Radix. Zhongguo Zhong Yao Za Zhi 40 (21), 4127–4135. 27071244

[B30] Zhang J. Hu X. Wang P. Huang B. Sun W. Xiong C. (2018). Investigation on Species Authenticity for Herbal Products of Celastrus Orbiculatus and Tripterygum Wilfordii from Markets Using ITS2 Barcoding. Molecules 23 (4). 10.3390/molecules23040967 PMC6017776 29690494

[B31] Zhao L. Yu X. Shen J. Xu X. (2018). Identification of Three Kinds of Plumeria Flowers by DNA Barcoding and HPLC Specific Chromatogram. J. Pharm. Anal. 8 (3), 176–180. 10.1016/j.jpha.2018.02.002 29922486 PMC6004628

[B32] Zhao X. Zhou Y. Wang L. Li M. Shi D. Li D. (2017). Shikonin Alleviates the Biotoxicity Produced by Pneumococcal Pneumolysin. Life Sci. 177, 1–7. 10.1016/j.lfs.2017.04.002 28385613

